# A Phthisical Bureau for London

**Published:** 1911-12-23

**Authors:** J. J. Perkins

**Affiliations:** Hon. Secretary. 20 Hanover Square. London, W.


*To the Editor of* The Hospital.

### A PHTHISICAL BUREAU FOR LONDON.

*To the Editor of* The Hospital.

Sir,—The National Association for the Prevention of Consumption is actively engaged in forming a library and bureau for the collection of all matters relating to pulmonary tuberculosis from every point of view, and in all countries. It is intended that such information shall be available not only to members of the medical profession, but to the public at large. Valuable assistance would be rendered if Medical Officers of Health, School Medical Officers, and Medical Superintendents or Secretaries of Hospitals, Sanatoria, Tuberculosis Dispensaries, and Open Air Schools would kindly place the Association on their distribution list in respect of annual reports or other documents bearing on the question of consumption. Books, pamphlets and reprints of articles from physicians and social workers in general would also be gladly received.—Believe me, yours very truly,

J. J. Perkins, Hon. Secretary.

20 Hanover Square.
London, W., December 16.

